# Mental well-being of Tunisian COVID-19 survivors: a cohort study

**DOI:** 10.1192/j.eurpsy.2024.1076

**Published:** 2024-08-27

**Authors:** M. Turki, N. Bouattour, H. Ben Ayed, S. Ellouze, R. Jbir, S. Msaad, S. Kammoun, N. Halouani, J. Aloulou

**Affiliations:** ^1^Psychiatry “B” department; ^2^Preventive medicine and hospital hygiene; ^3^Pneumology department, Hedi Chaker university hospital, Sfax, Tunisia

## Abstract

**Introduction:**

COVID-19 affected humankind worldwide in different aspects of life. Survivors still report the effects of the pandemic on daily life, physical health, and mental health.

**Objectives:**

To assess effects of the pandemic on the mood and the quality of life of the survivors.

**Methods:**

We conducted a prospective cohort study including 121 Tunisian COVID-19 inpatients who had been discharged alive from hospital. Each enrolled patient was asked about the period before the hospital stay, and the 6-9 month-period after hospital discharge, using several scales: the validated Arabic version of “Patient Health Questionnaire” (PHQ-9) to screen for depressive symptoms, and “EuroQol five-dimension three-level” (EQ-5D-3L) to assess the quality of life.

**Results:**

The median age of participants was 59 years, with extreme values ranging from 18 to 80. Among them, 51.2% were females. As compared with baseline statue of patients, the depressive dimension assessed through PHQ was significantly impaired (7.05 vs 1.12; p<0.001). The different dimensions of the EQ-5D-3L showed significant deterioration in mean scores (mobility:1.09 vs 1.31, p<0.001;  selfcare:1 vs 1.11, p=0.001; daily activities:1.09 vs 1.49, p<0.001; pain and disturbance: 1.17 vs1.49, p<0.0005 and anxiety and depression: 1.07 vs 1.57, p<0.001). Depressive symptoms were 10 times more frequent in post-COVID (57.9% vs 5.7%). The post-COVID PHQ-9 score was correlated with the post-COVID EQ-5D-3L score (p=0.033).

**Conclusions:**

This study points out the long-term impact of the COVID infection. Therefore, the clinician should screen for possible psychological distress even after resolution of the disease, in order to guarantee a better quality of life.

**Disclosure of Interest:**

None Declared

